# Medical and Physical Intelligence

**Published:** 1804-10-01

**Authors:** 


### MEDICAL AND PHYSICAL INTELLIGENCE.

### [ FOREIGN AND DOMESTIC. ]

---

### *Method of preparing Gallic Acid, by Mr. Fiedler.*

Boil one ounce of galls in sixteen ounces of water till it is reduced to eight ounces; separate the extractive matter from the acid by mixing with the liquor as much of pure argil as would make two ounces of sulphat of argil, and after some time filtrate the liquor. According to the author, the tannin, the extractive matter, and all the heterogeneous bodies, will remain on the filtre, combined with the argil, while the gallic acid is dissolved in the liquor that passes through.

---

Dr. KEUTSCH, a very able physician, resident in the Danish Island of Santa Cruz and St. Thomas, in the West Indies, has lately discovered a new method, and hitherto very successful, of treating the fevers of those Islands, so fatal to Europeans. His process process consists in frictions by oils. The first idea of this method he derived from the Theory of Dr. Scheel, of Copenhagen, on the use of oil in the plague: A theory which is to be found printed in the work of Baldwyn. Of eight soldiers that were entrusted to the care of Dr. Keutsch, six were happily delivered from the fever, at the end of twenty-four hours, by means of these frictions. They produced violent sweats, and always put a stop to vomiting. The doctor, in some particular cases, rendered the virtues of the oil still more efficacious, by adding camphor to it. This discovery is, of course, very valuable, as the fever cured is precisely the same as that which has made such cruel ravages in St. Domingo.

---

M. Tommasi, a Neopolitan chemist of some celebrity, who has been several years at Paris, has lately made many experiments to prove the power of the muriat of soda, or kitchen salt, in destroying the long white worms which are found in the intestinal canal. When he put those worms into a solution of an ounce of salt in fifty ounces of water, they did not live more than twenty-four minutes; but when the same quantity was dissolved in eight ounces of water, they lived only eight minutes. Hence he infers, that the method of curing this malady is easy and effectual.

---

Professor Paschmann, of St. Petersburgh, has invented an anemometer, by which the strength of the wind may be exactly measured; and, by means of other instruments, which are easily adjusted to it, such as a hygrometer, themometer, and barometer, a variety of physical experiments may be conducted with the greatest convenience.

---

A letter from the Honourable Frederick North, Governor of Ceylon, to the Right Honourable Lord Hobart, dated Jan. 1, 1804, received by Lord Camden, one of his Majesty's principal Secretaries of State, and communicated by his Lordship to the Royal Jennerian Society, contains the following information.

"Vaccination was unfortunately suspended, in some degree, while the English medical gentlemen attended the army at Kandy; and a spurious virus had been made use of in the northern district, the failures occasioned by which had discredited that beneficial practice. Genuine vaccine matter has, however, been sent thither, and confidence is restored throughout all these settlements, in that mode of inoculation. At Columbo, it is kept up with some difficulty, for want of subjects, as almost all the inhabitants of that neighbourhood have had the small-pox, in some manner or other; and the salutary consequences of the attention of government to that object, appears in the total absence of that disease from the province during the last six months; a circumstance hitherto unknown."

---

A Director General of Vacciolation has been appointed in the Italian Republic, to superintend all the inoculations of Cow-pock throughout throughout the Departments, the professional Members of which, are obliged to transmit him an account of their several proceedings.

---

A Case has occurred in Fullwood's Rents, Holborn, of supposed small-pox after vacciolation; but at a very large meeting of the profession this day, (Sept. 26) it was the general opinion that it was not small-pox. Most of the gentlemen who entertained the first idea, declined appearing; while the few who had considered the eruption as varicella, were confirmed in their conclusion by the observations of those who had not previously seen the case.

---

*Theatre, London Hospital.*

Mr. Headington and Mr. Frampton, will commence their First Course of Lectures upon Anatomy, Physiology, and the Principles and Operations of Surgery, on Monday, October 1, at two o'clock.

Demonstrations and Dissection as usual, by Mr. Armiger.

A series of Lectures upon Surgery, illustrated by Cases under Treatment, will be delivered during the winter by Sir William Blizard, Mr. Thomas Blizard, and Mr. Headington, surgeons of the Hospital.

On the 1st of October, also, Dr. Cooke will commence a course of Lectures upon the Practice of Physic; and Dr. Dennison upon the Theory and Practice of Midwifery.

---

Dr. Bradley's Lectures on the Practice of Medicine will commence on Friday the 5th of October next, at his house, No. 25, Parliament Street.

---

Dr. John Reid, senior Physician of the Finsbury Dispensary, intends to deliver, in the present winter, a Course of Lectures on the Theory and Practice of Medicine.

---

Mr. Thomas will commence his Lectures on the Principles and Operations of Surgery, on Monday, October 8, at his house in Leicester Square; where printed particulars may be had, and at the Anatomical Theatre in Windmill-street.

---

Mr. Moor, Surgeon Dentist to her Royal Highness the Duchess of York, will commence a Course of Lectures on the Structure and Diseases of the Teeth, on Monday the 5th of November; in which will be explained, the complete Practice of the Dentist.—Further particulars may be known by applying at his house, No. 6, Palsgrave Place, Temple.

